# CsGSTU8, a Glutathione S-Transferase From *Camellia sinensis*, Is Regulated by CsWRKY48 and Plays a Positive Role in Drought Tolerance

**DOI:** 10.3389/fpls.2021.795919

**Published:** 2021-12-09

**Authors:** Yongheng Zhang, Jingyuan He, Yezi Xiao, Yingao Zhang, Yingqin Liu, Siqing Wan, Lu Liu, Yuan Dong, Huan Liu, Youben Yu

**Affiliations:** College of Horticulture, Northwest A&F University, Xianyang, China

**Keywords:** *Camellia sinensis*, glutathione S-transferases (GSTs), ROS, WRKY TF, drought stress

## Abstract

Glutathione S-transferases (GSTs) constitute a large family of enzymes with a wide range of cellular functions. Recently, plant GSTs have gained a great deal of attention due to their involvement in the detoxification of electrophilic xenobiotics and peroxides under adverse environmental conditions, such as salt, cold, UV-B and drought stress. A previous study reported that a GST gene (*CsGSTU8*) in tea plant was distinctly induced in response to drought, suggesting this gene plays a critical role in the drought stress response. In this study, by using quantitative real-time PCR (qRT-PCR) and β-glucuronidase (GUS) reporter lines, we further demonstrated that *CsGSTU8* was upregulated in response to drought stress and exogenous abscisic acid (ABA) treatments. Overexpression of *CsGSTU8* in *Arabidopsis* resulted in enhanced drought tolerance as indicated by the improved scavenging of excess amounts of reactive oxygen species (ROS) under drought conditions. Furthermore, we found that CsWRKY48 acts as a transcriptional activator and that its expression is induced in response to drought stress and ABA treatment. Electrophoretic mobility shift assays (EMSAs), dual-luciferase (LUC) assays and transient expression assays in tea plant leaves revealed that CsWRKY48 directly binds to the W-box elements in the promoter of *CsGSTU8* and activates its expression. Taken together, our results provide additional knowledge of drought stress responses in tea plant.

## Introduction

Adverse environmental conditions, especially drought conditions, greatly limit plant growth and development (Shao et al., 2008). Plants have evolved intricate defensive systems to survive under drought-stress conditions, specifically, regulating the balance of reactive oxygen species (ROS) (including hydrogen peroxide (H_2_O_2_), superoxide (O^2–^), OH^•^ and ^1^O_2_) in cells is an indispensable strategy (Carvalho, 2008; Singh et al., 2019). On the one hand, ROS are necessary for plant growth, as plants actively produce ROS that serve as signal transduction molecules for growth under suitable conditions. On the other hand, when plant experience drought stress, excessive amounts of ROS accumulate in cells and cause irreversible damage to membranes, proteins, and RNA and DNA molecules, even resulting in oxidative destruction of the cells (Moller et al., 2007; Choudhury et al., 2017). Thus, accelerating the expression or activities of antioxidant enzymes and antioxidants, which are involved in ROS scavenging, is critical for the drought tolerance of plants (Smirnoff, 1993; Gill and Tuteja, 2010; Qi et al., 2018).

Glutathione S-transferases (GSTs) constitute a large family of enzymes with a wide range of cellular functions in plants, including protecting organisms against oxidative stress under stress conditions by participating in ROS scavenging (Jha et al., 2011; Rong et al., 2014; Qi et al., 2018). Plant GSTs can be divided into eight distinct subclasses according to their protein sequence and function: Phi (GSTF), Tau (GSTU), Theta (GSTT), Zeta (GSTZ), Lambda (GSTL), Elongation factor 1 gamma (EF1G), DHAR, and TCHQD proteins (Sasan et al., 2011). Recently, several classes of GSTs, including Tau GSTs, have received a great deal of attention in plants due to the assistance of these GSTs in regulating the metabolism of oxidized molecules under drought conditions (Liu et al., 2013b; Xu et al., 2016; Choudhury et al., 2017). The expression level of *GSTs* in plants was reported to be positively correlated with the rate of oxidized molecule scavenging and to contribute to drought stress tolerance in previous studies (Kumar et al., 2013; Stavridou et al., 2021). For example, transgenic *Arabidopsis* plants overexpressing tomato *LeGSTU2* show enhanced resistance to drought stress via increased antioxidative enzyme activities for scavenging excess ROS (Xu et al., 2015). Liu et al. (2013a) reported that overexpression of a zeta GST gene from *Pyrus pyrifolia* in tobacco improved O^2–^ scavenging under drought conditions, thus resulting in increased tolerance to drought. Moreover, overexpression of a GST gene (*ThGSTZ1*) from *Tamarix hispida* improves drought tolerance by enhancing the ability to scavenge ROS (Yang et al., 2014).

Although *GSTs* have been observed to protect cells from oxidative stress under drought-stress conditions, knowledge about the intricate regulation of *GSTs* under drought stress remains scarce. To date, several *GSTs* have been reported to be directly activated by specific transcription factors to regulate drought stress tolerance. For example, in wheat, the BES/BZR family transcription factor TaBZR2 functions positively in the drought response by activating *TaGST1* to scavenge drought-induced O2^–^ accumulation (Cui et al., 2019). A wheat ethylene-response factor, TaERF3, was also reported to directly activate the expression of *TaGST6* by binding to the *TaGST6* promoter to improve drought tolerance (Rong et al., 2014). Furthermore, Ren et al. (2020) demonstrated that a NAC transcription factor, ZmNST3, enhances maize drought stress tolerance by directly binding to the promoters of *GST* and *GlnRS* and activating their expression.

*Camellia sinensis* is a commercially important perennial evergreen woody crop species that is widely cultivated worldwide and is highly susceptible to drought stress (Parmar et al., 2019). Although several tea plant *GSTs* have been reported to be involved in cold and drought stress responses based on their expression levels (Parmar et al., 2019; Samarina et al., 2020; Sun et al., 2020), the roles of tea plant *GSTs* in drought stress are still poorly understood. *CsGSTU8* was found to be strongly induced in response to drought stress in previous studies (Xia et al., 2019) and according to our transcriptome data (unpublished), thus prompting us to investigate its role in drought stress. In this study, we used quantitative real-time PCR (qRT-PCR) and transgenic β-glucuronidase (GUS) reporter lines to confirm the upregulation of *CsGSTU8* under drought and abscisic acid (ABA) treatments. Furthermore, transgenic *Arabidopsis* plants expressing *CsGSTU8* showed improved ROS scavenging, thus contributing to drought resistance. In addition, CsWRKY48, a tea plant transcriptional activator, whose expression is induced in response to drought and ABA treatment, can directly bind to the promoter and activate the expression of *CsGSTU8*. Taken together, our findings provide evidence for the positive role of the GSTU8 protein in drought stress and are helpful for understanding the mechanism of the drought response in tea plant.

## Materials and Methods

### Plant Materials and Treatment

Hydroponically grown Longjing-changye annual tea seedlings were preincubated in a greenhouse at Northwest A&F University Yangling (34°20′N, 108°24′E), Shaanxi Province, China, under natural light, and the temperature was maintained at 20/25°C in the dark/light. Seedlings displaying consistent growth were divided into three treatment groups: normal, 15% polyethylene glycol (PEG) 6,000 and ABA (100 μM) treatments. The first to third leaves were collected at 0, 4, 8, 12, 24, and 48 h after treatment for RNA extraction. For all the treatments, three biological replicates were included.

### RNA Extraction and qRT-PCR Analysis

Total RNA extraction and qRT-PCR were carried out as previously described by Wan et al. (2020). The primers used in the expression analysis are listed in Supplementary Table 1.

### Transformation of *Arabidopsis* With *CsGSTU8* and Subcellular Localization of CsGSTU8

The CDS of *CsGSTU8* was amplified from the cDNA of Longjing-changye tea plant according to the sequence information reported in the Tea Plant Information Archive (TPIA) (Xia et al., 2019). Then, the Kpn I and BamH I sites of pCAMBIA2300-GFP vector were selected to generate 35S::CsGSTU8-GFP construct. To obtain transgenic *Arabidopsis*, *Agrobacterium tumefaciens* strain GV3101 harboring the 35S::CsGSTU8-GFP construct was transformed into wild-type (WT) *Arabidopsis* ecotype Columbia (Col-0) by using the floral-dip method (Zhang et al., 2006). T3-generation homozygous lines (OE3 and OE7) and Col-0 (WT) plants were used for further analysis.

To determine the subcellular localization of the CsGSTU8 protein, GV3101 cells harboring *35S*::CsGSTU8-GFP and *35S*::GFP were cultured and adjusted to an OD600 of 0.6 via an infiltration buffer (10 mM MES, 10 mM MgCl_2_ and 100 μM acetosyringone; pH 5.7). After 2 h incubation at 25°C, the cultures were transiently expressed in 4-week-old tobacco (*Nicotiana benthamiana*) leaves, and the GFP signal was observed with a BX63 Automatic Fluorescence Microscope (Olympus, Japan) at 48 h after infiltration. The primers used are listed in Supplementary Table 1.

### Stress Tolerance Assays

Transgenic and WT *Arabidopsis* seeds were surface sterilized with 20% NaClO (including 0.1% Triton X-100) and sown on Murashige and Skoog (MS) agar media that included mannitol (0, 100, 125, and 150 mM). Media containing seeds were placed in a controlled growth chamber under 16/8 h (25/20°C) day/night conditions for growth after incubation at 4 °C for 3 days. The survival rate was calculated after 6-days (normal conditions) or 10-days (mannitol conditions) of growth. Seedlings with green leaves under mannitol conditions were considered alive.

For drought treatment in soil, WT and transgenic seeds were germinated in soil (vermiculite: humus = 1:1) and grown under normal conditions for 7 days. Then watering was withheld until drought stress symptoms occurred. Relative electrolyte leakage was measured to compare the drought tolerance of WT and transgenic seedings following previously report (Jiang et al., 2019).

### Measurement of Reactive Oxygen Species Contents, Malondialdehyde Contents and Glutathione S-Transferase Activity

3,3′-Diaminobenzidine (DAB) staining and nitro blue tetrazolium (NBT) staining were performed to detect the accumulation of H_2_O_2_ and O^2–^ in the leaves according to our previously described methods (Zhang et al., 2020). The contents of H_2_O_2_ and O^2–^ were measured following the previous report (Shi et al., 2010). To determine the Malondialdehyde (MDA) content, a thiobarbituric acid (TBA) reaction was performed according to a previously described method (Draper et al., 1992). The GST activity was measured according to the protocol provided with a glutathione-S-transferase Assay Kit (BCO350, Solarbio, China).

### β-Glucuronidase Reporter Construction and β-Glucuronidase Activity Assays

The 1,500 bp promoter of *CsGSTU8* was inserted into a pCAMBIA1300-GUS vector (Hind III and BamH I) to activate the GUS gene. Then, the construct was transformed into WT *Arabidopsis*; 7-day-old T2-generation seedings were treated with ABA (1 μM) and dehydrated (20% PEG 6,000). After 12 h of treatment, GUS staining and activity were measured as described previously (Jefferson et al., 1987). The primers used are listed in Supplementary Table 1.

### Electrophoretic Mobility Shift Assays

Full-length CsWRKY48 (TEA008513) was inserted into a pGEX4T-1 vector to construct a GST-CsWRKY48 expression vector, and the recombinant plasmid was transformed into *E. coli* strain Rosetta2 (EC1014, Weidi Biotechnology, Shanghai, China). Expression of the GST and GST-CsWRKY48 proteins was induced by 1 mM isopropyl-b-D-thiogalactoside (IPTG) at 16°C for 20 h, and then the fusion proteins were purified using glutathione Sepharose (GSTrap™ HP, GE Healthcare, United States). A biotin-labeled probe containing “W-box” elements in the promoter of *CsGSTU8* was synthesized by Tsingke. EMSAs were carried out according to the protocol provided with a chemiluminescent EMSA kit (GS009, Beyotime Biotechnology, China).

### Transient Transcriptional Activation Assays

The 1,500 bp promoter fragment of *CsGSTU8* was inserted into the luciferase (LUC) reporter plasmid pGreen II 0800, which contains a Renilla luciferase (REN) gene under the control of the *35S* promoter used as an internal control. The *35S*::CsWRKY48-GFP vector as effector. The effector plasmids and the reporter plasmids were transformed into GV3101 (pSoup), and the effector and corresponding reporter in GV3101 (pSoup) were mixed together at a proportion of 2:1 and subsequently injected into tobacco leaves. Forty-eight hours later, the LUC signal was visualized with a CCD system (Lumazone Pylon 2048B, Princeton, United States). The activities of LUC and REN were determined according to the protocol of a dual-Luciferase reporter assay kit (FR201, TransGen Biotech, China) by using a full-wavelength multifunctional enzyme labeling instrument (Tecan Infinite M200PRO, Tecan, Switzerland).

Two-month-old Fudingdaba tea plants grown from seeds in a growth chamber were selected for transient expression in accordance with the methods of Shui et al. (2021) with slight modifications. *A. tumefaciens* strain GV3101 harboring *35S*::CsWRKY48-GFP and *35S*::GFP was injected into the different sides of the same leaf. At 3 days after injection, leaves were collected for RNA isolation, and RT-PCR and qRT-PCR were then carried out to assess the transformation results and the measure expression of *CsGSTU8*, respectively. The primers used are listed in Supplementary Table 1.

### Statistical Analysis

SPSS 19.0 software was used for statistical data analysis. Data for tissue-specific expression, survival rate and relative electrolyte leakage were subjected to one-way analysis of variance, and the differences between means were assessed by Duncan’s multiple range tests. Other data were examined by Student’s *t*-tests. The values are represented as means ± standard deviations.

## Results

### Identification, Expression in Different Tissues and Subcellular Localization of CsGSTU8

**FIGURE 1:**
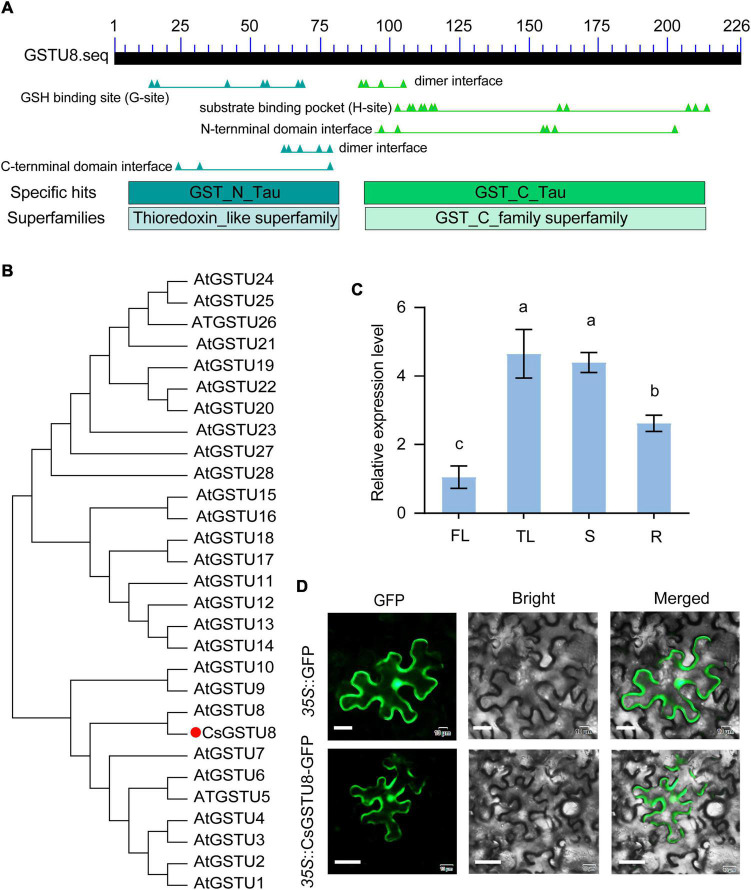
Characterization of CsGSTU8 from *Camellia sinensis*. **(A)** Conserved domain analysis of the CsGSTU8 protein sequence retrieved from the NCBI database (https://www.ncbi.nlm.nih.gov/Structure/cdd/). **(B)** Phylogenetic analysis of CsGSTU8 and GSTU proteins from *Arabidopsis*. **(C)** Expression of CsGSTU8 in different tissues. FL (first leaf), TL (third leaf), S (stems), R (root tips). The data are the means ± SDs of three independent experiments. The values not followed by the same letter are significantly different according to Duncan’s multiple range test (*P* < 0.05). **(D)** Subcellular localization of 35S::CsGSTU8-GFP and 35S::GFP in tobacco cells,. Bar = 20 μm.

Our transcriptome data (Supplementary Figure 1) and those from the TPIA^1^ revealed that TEA019065 was strongly responsive to drought stress (Xia et al., 2019). In addition, it was also reported to be distinctly induced in response to drought stress in both drought-tolerant and drought-sensitive tea plants (Parmar et al., 2019). These findings prompted us to investigate the role of this gene in drought stress. Therefore, we isolated the complete CDS of TEA019065 from Longjing-changye seedings and found that it encodes a GST belonging to the Tau subfamily and harbors a GSH-binding site in the N-terminal region (Figure 1A). The protein comprises 226 amino acids and has a molecular weight of 26.36 kDa. Phylogenetic analysis including all Tau GST proteins from *Arabidopsis* showed that TEA019065 is closely related to AtGSTU8 (Figure 1B); hence, it was named CsGSTU8 in this study.

To explore the expression levels of *CsGSTU8* in different tissues, we investigated its expression in the first leaves, third leaves, stems and root tips via qRT-PCR. The results showed that *CsGSTU8* tended to expressed greater in the mature organization (the third leaves and stem) than young organs (the first leaves and root tips) (Figure 1C).

*A. tumefaciens* strain GV3101 harboring *35S*::CsGSTU8-GFP and *35S*::GFP was introduced into tobacco leaves to investigate the subcellular localization of the CsGSTU8 protein. The fluorescence of GFP fused to CsGSTU8 showed no difference from that of the GFP control, which means that the CsGSTU8 protein was distributed throughout the cell (Figure 1D).

### Expression Analysis of *CsGSTU8* Under Drought and Abscisic Acid Treatments

**FIGURE 2:**
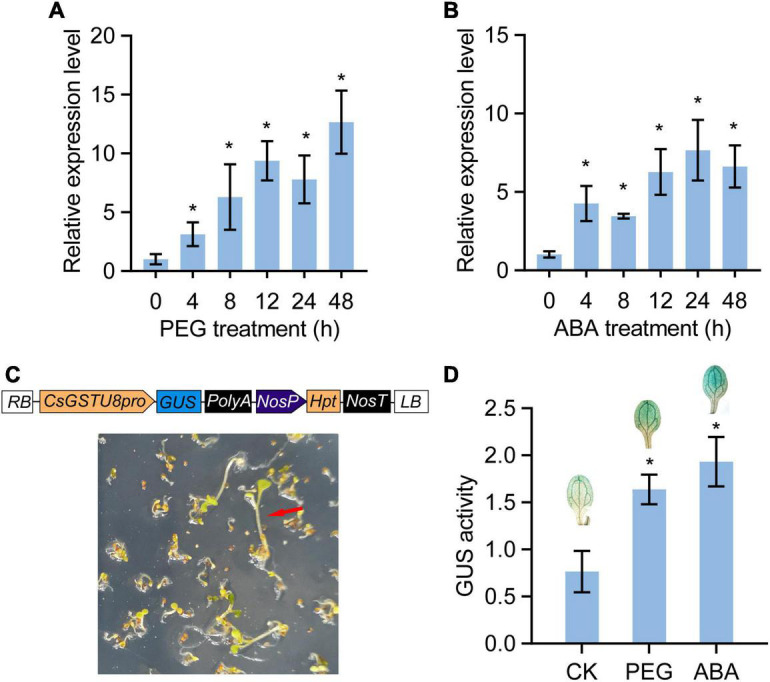
Expression patterns of *CsGSTU8* under PEG and ABA treatments. **(A)** Expression of *CsGSTU8* after PEG treatment. **(B)** Expression of *CsGSTU8* under ABA treatment. **(C)** Construction of *CsGSTU8pro*::GUS transgenic lines. Hygromycin B was used to screen transgenic *Arabidopsis* plants. **(D)** Measurement of the GUS activities in *CsGSTU8pro*::GUS transgenic *Arabidopsis* leaves under PEG and ABA treatments. The Data are presented as the means ± SDs of three independent experiments. Significant differences were determined using Student’s *t*-test (*P < 0.05).

To validate the drought response of *CsGSTU8*, tea plants were treated with 15% PEG 6,000 to mimic drought stress, and the transcript levels of *CsGSTU8* were measured by qRT-PCR. The results showed that the expression of *CsGSTU8* gradually increased in response to drought stress (Figure 2A). Notably, ABREs involved in ABA responsiveness were found in the promoter of *CsGSTU8* (Supplementary Figure 2), implying that the *CsGSTU8* expression is induced in response to ABA treatment. qRT-PCR was used to measure the expression of *CsGSTU8* under ABA treatment, and as expected, ABA induced the expression of *CsGSTU8* (Figure 2B). To better understand *CsGSTU8* expression patterns, a *CsGSTU8pro*::GUS transgenic line was generated and subjected to drought and ABA; the results showed that GUS activity significantly increased under drought stress and ABA treatment (Figures 2C,D). Taken together, these results further reveal that the expression of *CsGSTU8* was induced in response to drought stress and ABA treatment.

### *CsGSTU8* Confers Drought Tolerance to Transgenic *Arabidopsis* Plants

**FIGURE 3:**
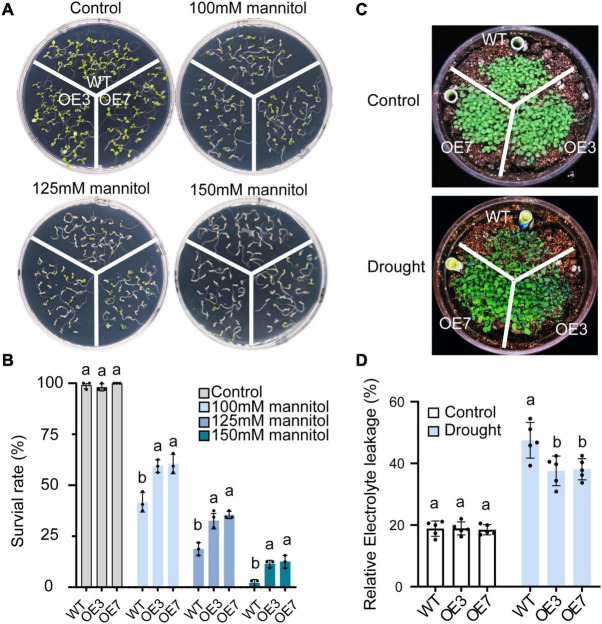
Overexpression of *CsGSTU8* improves drought stress tolerance in transgenic *Arabidopsis*. **(A)** Growth phenotypes under control and mannitol conditions in MS media. **(B)** Measurement of survival rate in MS media. The Data are presented as the means ± SDs of three independent experiments. **(C)** Growth phenotypes under control and drought stress conditions in the soil. **(D)** Measurement of relative electrolyte leakage. The Data are the means ± SDs of five independent experiments. The Values not followed by the same letter are significantly different according to Duncan’s multiple range test (*P* < 0.05).

Transgenic *Arabidopsis* plants were generated to explore the function of *CsGSTU8* in tolerance to drought stresses. Two T3-generation homozygous lines (OE3 and OE7) with high expression of *CsGSTU8* gene were selected for further analysis (Supplementary Figure 3). Various concentrations of mannitol were employed to mimic drought stress. As expected, in the absence of mannitol condition, the survival rates of the two transgenic lines were indistinguishable from those of the WT (Figures 3A,B). The application of mannitol inhibited the growth of both the WT and transgenic lines in a dose-dependent manner; however, the survival rates of the OE3 and OE7 lines significantly increased compared to those of the WT plants under 100, 125, and 150 mM mannitol conditions (Figures 3A,B).

Moreover, under soil water shortage conditions, the WT plants exhibited a more severely wilted phenotype than did the OE3 and OE7 transgenic lines (Figure 3C). We further measured the relative electrolyte leakage to evaluate membrane damage in *Arabidopsis* leaves, and the results showed that there was no difference between the WT and transgenic plants under normal conditions, while under drought stress, the relative electrolyte leakage of the two transgenic lines was lower than that of the WT (Figure 3D). These results indicate that overexpression of *CsGSTU8* enhances drought tolerance in *Arabidopsis*.

### Overexpression of *CsGSTU8* Alleviates Reactive Oxygen Species and Malondialdehyde Accumulation in *Arabidopsis*

**FIGURE 4:**
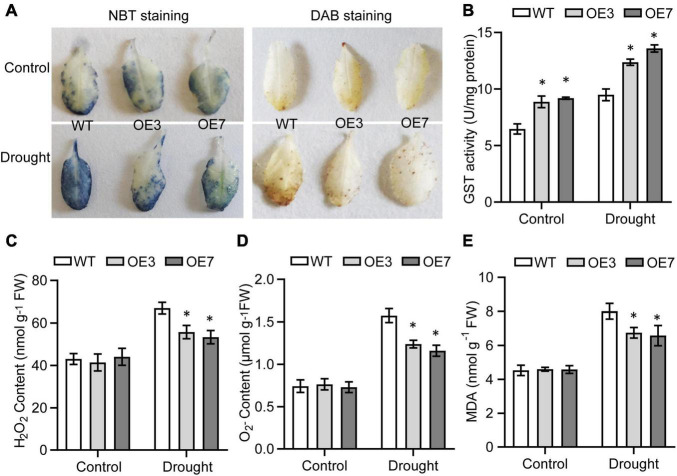
Overexpression of *CsGSTU8* alleviated ROS and MDA accumulation under drought stress. **(A)** Histochemical detection of O^2–^ and H_2_O_2_ via NBT staining and DAB staining, respectively. **(B)** Measurement of GST activity under control and drought conditions. **(C)** Measurement of H_2_O_2_ content under control and drought conditions. **(D)** Measurement of O^2–^ content under control and drought conditions. **(E)** Measurement of MDA content under control and drought conditions. The Data are presented as the means ± SDs of three independent experiments. Significant differences were determined using Student’s *t*-test (*P < 0.05).

As it is well established that GSTs contribute to alleviating excess amounts of ROS when plants experience stress (Sharma et al., 2014; Yang et al., 2014), we evaluated the GST activity and ROS content to explore whether enhanced drought tolerance is associated with a decrease of ROS levels in transgenic lines. The results showed that GST activity was higher in the transgenic plants than in the WT plants under both control and drought conditions (Figure 4B). Interestingly the contents of H_2_O_2_ and O^2–^ in the WT and transgenic lines were not significantly different under normal conditions, while under drought stress, the OE3 and OE7 transgenic lines accumulated less H_2_O_2_ and O^2–^ than the WT plants did (Figures 4A,C,D). MDA is considered a marker of membrane lipid peroxidation caused by excessive ROS (Jambunathan, 2010), so we also measured the MDA content of WT and transgenic lines under drought stress. The transgenic lines consistently accumulated lower MDA than did the WT under drought conditions (Figure 4E). These results implying that *CsGSTU8* overexpression in *Arabidopsis* alleviates ROS and MDA accumulation under drought stress.

### CsWRKY48 Is a Transcriptional Activator Involved in Responses to Drought and Abscisic Acid in Tea Plant

**FIGURE 5:**
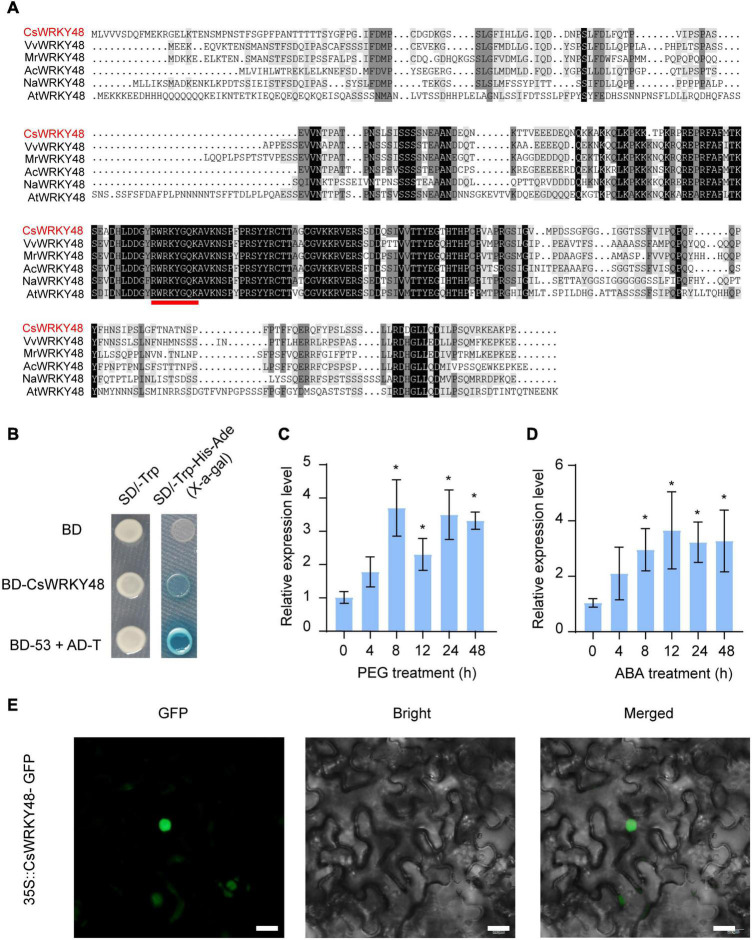
CsWRKY48 acts as a transcriptional activator and is expressed in response to drought and ABA treatment. **(A)** Multiple sequence alignment of CsWRKY48 and its homologs in other plant species. The red line indicates the WRKY DNA-binding domain. **(B)** Transactivation analysis of CsWRKY48 in yeast. **(C)** Transcript levels of *CsWRKY48* under PEG treatment. **(D)** Transcript levels of *CsWRKY48* under ABA treatment. **(E)** Subcellular localization of 35S::CsWRKY48-GFP in tobacco cells. Bar = 20 μm. The data are presented as the means ± SDs of three independent experiments. Significant differences were determined using Student’s *t*-test (*P < 0.05).

Our team previously reported that *CsWRKYIIc3* (annotated as *CsWRKY48* in the TPIA) is involved in responses to drought and ABA in tea plant Shaancha No. 1 tea plant (Xiao et al., 2020). Our unpublished transcriptome data also demonstrated that *CsWRKY48* expression was induced in response to drought (Supplementary Figure 1). In this study, qRT-PCR was used to explore whether *CsWRKY48* responds to drought and ABA treatment in Longjing-changye seedings, and the results showed that *CsWRKY48* was indeed induced in responses to drought and ABA treatment (Figures 5C,D). Amino acid sequence analysis and subcellular localization results revealed that CsWRKY48 contains a highly conserved WRKY DNA-binding domain (Figure 5A) and localizes to the nucleus (Figure 5E), so we fused the CDS of *CsWRKY48* with the Gal4-binding domain in the pGBKT7 vector, after which the construct was transferred to yeast strain Y2H Gold to investigate its transactivation capacity. The results showed that the fusion of CsWRKY48 with Gal4 was able to activate *HIS3*, *ADE2* and *MEL1* and grow on selective media (Figure 5B), indicating that CsWRKY48 has potential transcriptional activation.

### CsWRKY48 Bind to the *CsGSTU8* Promoter and Activates the Expression of *CsGSTU8*

**FIGURE 6:**
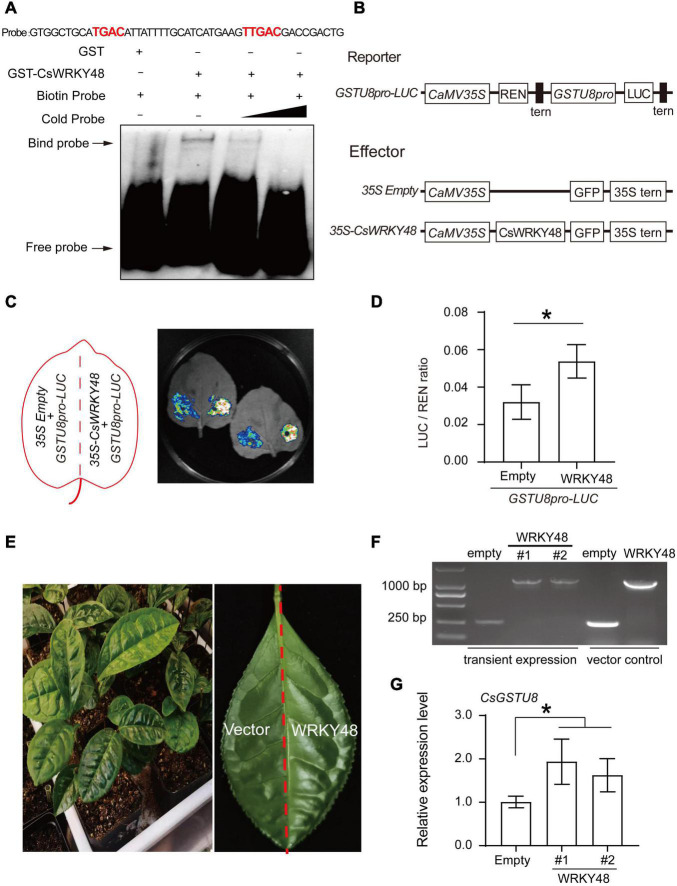
CsWRKY48 binds to the promoter of *CsGSTU8* and activates its expression. **(A)** EMSAs using purified GST and GST-CsWRKY48 proteins incubated together with biotin-labeled probes of W-box core elements (TGAC sequences) presented in *CsGSTU8* promoter. -: absence; +: presence. **(B)** Schematics of the reporter and effector constructs used in the dual-LUC assays. **(C)** LUC signal detected in tobacco leaves. **(D)** LUC/REN ratio, as measured by the dual-LUC assays. **(E)** Transient expression of 35S::CsWRKY48-GFP and 35S::GFP in tea plant leaves. **(F)** RT-PCR was used to identify the transformation of 35S::CsWRKY48-GFP and 35S::GFP by using specific primers. **(G)** Expression of *CsGSTU8* in transformed tea plant leaves. The data are presented as the means ± SDs of three independent experiments. Significant differences were determined using Student’s *t*-test (*P < 0.05).

W-boxes were found in the promoter of *CsGSTU8* (Supplementary Figure 2), implying that the expression of *CsGSTU8* might be regulated by WRKY transcription factors. Remarkably, our results revealed that the expression of *CsGSTU8* similar to that of *CsWRKY48*, was induced in response to both drought and ABA treatment, and we inferred that CsWRKY48 may be involved in the regulation of *CsGSTU8* expression. Thus, EMSAs and dual-LUC transient expression assays were carried out to verify this hypothesis. First, GST and GST-CsWRKY48 were purified for use in EMSAs and the band shifts were observed when the GST-CsWRKY48 protein was incubated together with a biotin-labeled probe (containing the core sequences (TGAC) of W-boxes), while band shifts were not observed in the GST protein control (Figure 6A), which indicated that CsWRKY48 can directly bind to the *CsGSTU8* promoter. Dual-LUC transient expression assays showed that the transient expression of *CsWRKY48* significantly increased the LUC/REN ratio of the reporter relative to that of the corresponding empty control in tobacco leaves (Figures 6B–D). In addition, we transiently overexpressed *CsWRKY48* in tea plant leaves (Figures 6E,F), and the qRT-PCR results showed that *CsWRKY48* overexpression increased the expression of *CsGSTU8* in tea plant (Figure 6G). Taken together, our results indicate that CsWRKY48 can directly bind to the promoter of *CsGSTU8* and activate its expression.

## Discussion

GSTs with a wide range of functions in plants, including protecting cells from oxidative damage during stresses (Sheehan et al., 2001). A series of studies have indicated that the expression of GST genes is induced in response to drought stress in various plants, including barley (Rezaei et al., 2013), tomato (Xu et al., 2015), wheat (Wang et al., 2019), potato (Islam et al., 2018), pepper (Islam et al., 2019), and rice (Jain et al., 2010). Previous studies in tea plant have reported that drought stress also induces the expression of members of *CsGSTs* (Parmar et al., 2019; Xia et al., 2019; Sun et al., 2020), including *CsGSTU8*, implying that *GSTs* from tea plant may have conserved functions in response to drought stress, similar to cases in other plants. Based on this information, we investigated the role and regulation of *CsGSTU8* in response to drought stress in this study.

As it well established that abiotic stress, such as drought, salinity and cold alter the ABA levels in plants. ABA plays an important role as an essential mediator in triggering plant responses to various abiotic stresses (Hartung et al., 1988). Several *GSTs* have been reported to be associated with ABA signaling and to be involved in the stress response. For example, Sharma et al. (2014) reported that *OsGSTU4* was induced in response to ABA and was involved in ABA-dependent processes that provide stress tolerance to transgenic plants. In wheat, the TaERF3-actived *TaGST6* expression was enhanced by ABA to respond to salt and drought stress (Rong et al., 2014). Our results indicated that the expression of *CsGSTU8* was increased in response to ABA and drought treatments, meanwhile, the ABA- and drought-responsive transcription factor CsWRKY48 could directly activate the expression of *CsGSTU8*. Thus, we inferred that ABA signaling might be involved in the drought-induced expression of *CsGSTU8*.

One of effects of drought stresses on plants is the excess accumulation of ROS. A series of studies have shown that transgenic plants expressing *GSTs* contribute to drought stress tolerance by scavenging excess ROS accumulation (Liu et al., 2013a; Srivastava et al., 2019; Yang et al., 2019). Similarly, in the current study, transgenic *Arabidopsis* plants expressing *CsGSTU8* exhibited a stress-tolerant phenotype when subjected to drought stress accompanied by less accumulation of ROS level, suggesting the positive role of CsGSTU8 in ROS scavenging. GST proteins catalyze the conjugation of GSH to an array of hydrophobic and electrophilic substrates, including ROS, thus, protecting the cell from oxidative burst (Kumar and Trivedi, 2018). During catalysis, the binding and correct orientation of GSH are governed by the conserved GSH binding site (G-site), and the substrate binding pocket (H-site) assists in the binding of substrates by providing a hydrophobic environment (Basantani and Srivastava, 2007; Kumar and Trivedi, 2018). CsGSTU8 harboring the conserved GSH bind site and the substrate binding pocket, suggesting the capacity of CsGSTU8 to catalyze the conjugation of GSH to an array of substrates, which was further confirmed by the higher GST activity of transgenic plants compared to that in WT plants. Thus, the lower ROS level of transgenic plants under drought stress can be explained by the fact that transgenic plants have an improved capacity for GSH conjugation to ROS and result in an enhanced ability to scavenge ROS, which constitutes the foundation for their stress-tolerant phenotype.

During signal transduction and the adaptive response, transcription factors including NAC, MYB, bZIP, DREB, and WRKY ones usually bind to the promoters of effector genes that encode enzymes, chaperones and ion/water channels and directly regulate their expression to induce an adaptive response under drought conditions (Joshi et al., 2016; Wei et al., 2019). As effector genes encoding enzymes involved in ROS scavenging under stress, *GSTs* have been found to be directly regulated by several type transcription factors in response to drought stress in plants, such as ERF type (Rong et al., 2014), bHLH type (Cui et al., 2019), and NAC type (Ren et al., 2020). WRKY family members regulate the expression of target genes by binding to the W-box cis-elements in their promoters (Deng et al., 2020; Zhao et al., 2020). For example, AtWRKY57 can directly bind to the W-box in *AtRD29A* and *AtNCED3* promoters and activate their expression (Jiang et al., 2012). Our EMSA and Dual-LUC transient expression assays demonstrated that a drought- and ABA-responsive transcription factor form tea plant, CsWRKY48, can directly bind to the W-box in promoter of *CsGSTU8* and activate its expression. These results suggest that drought stress may first induces the expression of *CsWRKY48* and then activates the *CsGSTU8* transcription, thus causing an adaptive response.

## Conclusion

In conclusion, our study revealed that *CsGSTU8*, which is positively activated by CsWRKY48, enhances the drought tolerance of transgenic *Arabidopsis* by increasing ROS scavenging under drought stress. This study provides valuable knowledge for understanding the function of CsGSTU8 and the underlying molecular mechanism of drought tolerance in tea plant.

## Data Availability Statement

The original contributions presented in the study are included in the article/Supplementary Material, further inquiries can be directed to the corresponding author/s.

## Author Contributions

YY and YoZ designed the experiments. YoZ and JH performed the experiments and data analysis. YoZ and YX wrote the manuscript. SW, YiZ, and YL provided valuable advice on the manuscript. LL, YD, and HL revised the manuscript. All authors contributed to the article and approved the submitted version.

## Conflict of Interest

The authors declare that the research was conducted in the absence of any commercial or financial relationships that could be construed as a potential conflict of interest.

## Publisher’s Note

All claims expressed in this article are solely those of the authors and do not necessarily represent those of their affiliated organizations, or those of the publisher, the editors and the reviewers. Any product that may be evaluated in this article, or claim that may be made by its manufacturer, is not guaranteed or endorsed by the publisher.

## Footnotes

1
http://tpia.teaplant.org/
