# Supplementary material for: CsGSTU8, a Glutathione S-Transferase From Camellia sinensis, Is Regulated by CsWRKY48 and Plays a Positive Role in Drought Tolerance
Source: Front Plant Sci. 2021 Dec 9;12:795919. doi: 10.3389/fpls.2021.795919 (PMC8696008; doi:10.3389/fpls.2021.795919)
Supplement: Supplementary file 1 [file Data_Sheet_1.docx]

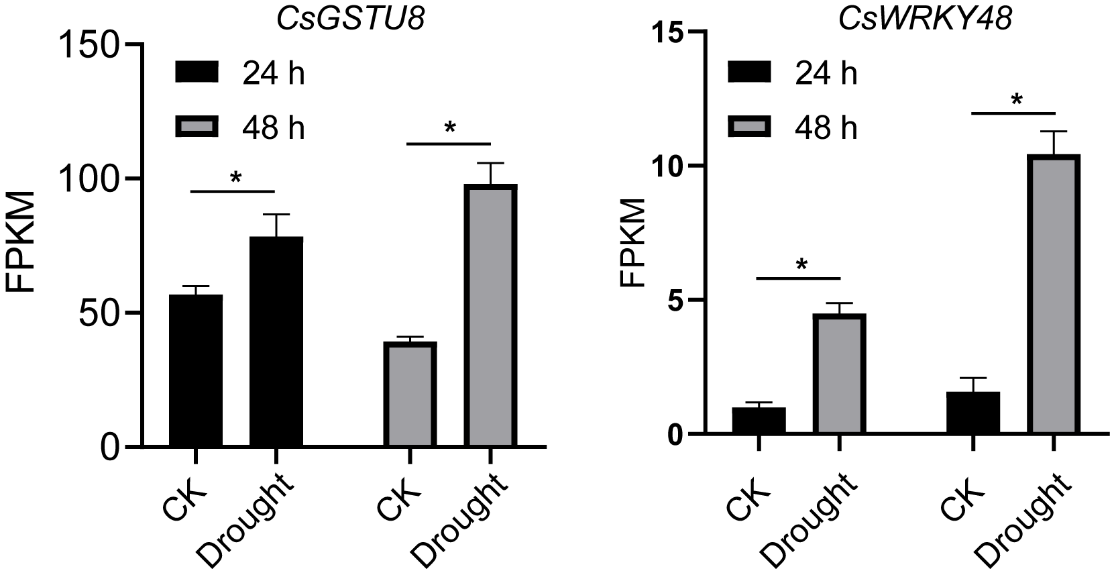


**Fig.S1. Transcript level of *CsGSTU8* and *CsWRKY48* in our unpublished transcriptome data.** Data are presented as the means ± SDs of three independent experiments. Significant differences were determined using Student’s t-test (*, P < 0.05).


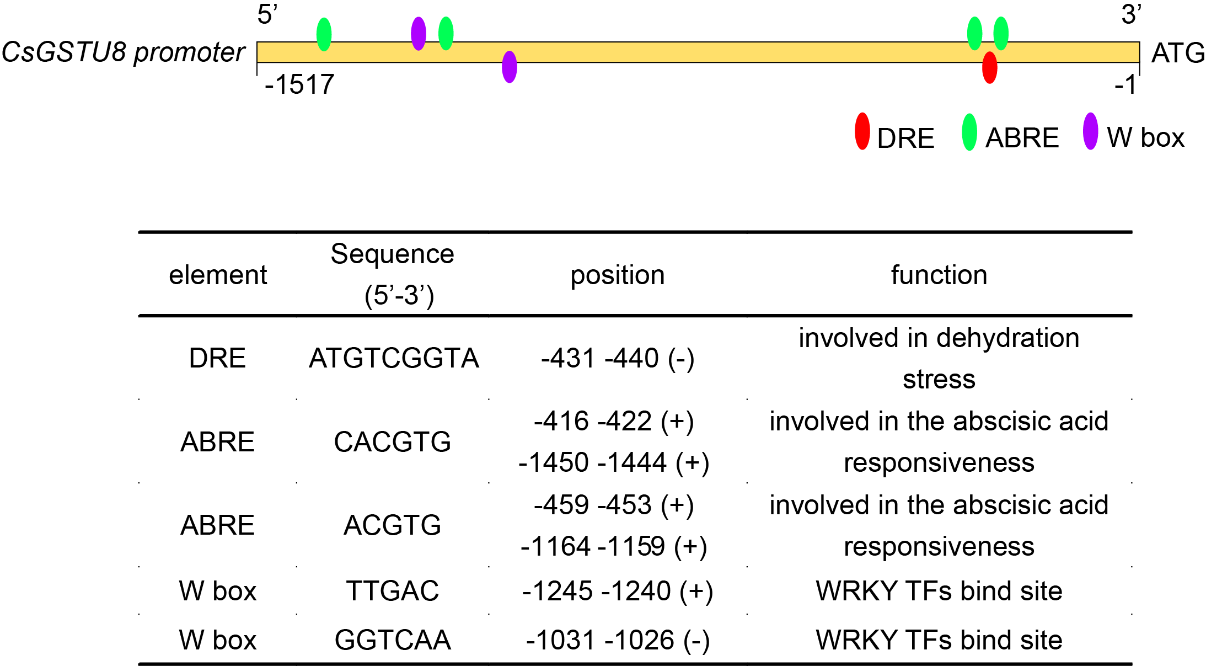


**Fig.S2. *Cis*-elements identification of *CsGSTU8* promoter in PlantCARE** (<http://bioinformatics.psb.ugent.be/webtools/plantcare/html/>).


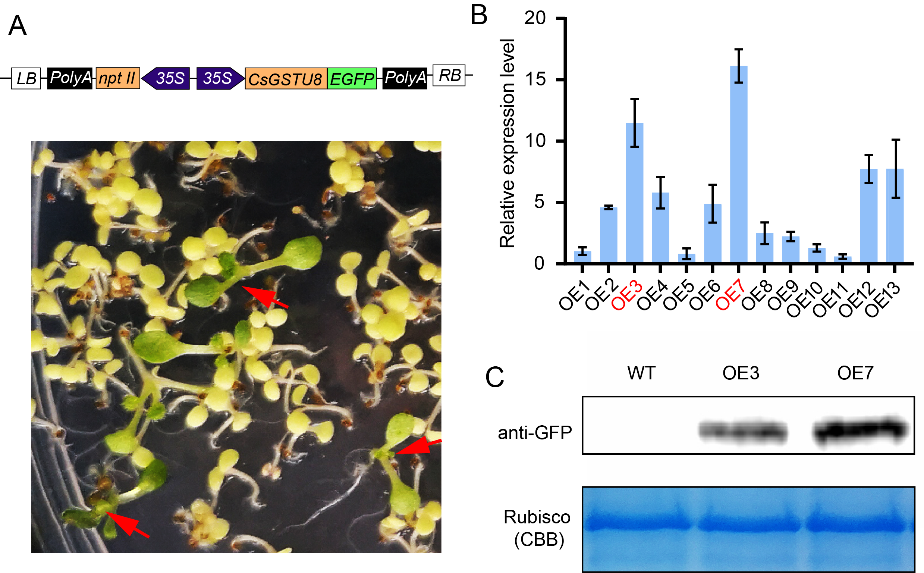


**Fig.S3. Identification of *CsGSTU8* transgenic *Arabidopsis*.** (A) Screening of transgenic plants with kanamycin resistance. (B) Expression level analysis of *CsGSTU8* in transgenic lines by using qRT-PCR, red marked lines were used in this study. (C) Western blot analysis of CsGTU8-GFP fusion protein in transgenic *Arabidopsis* using GFP antibody.
